# Salting-out assisted liquid–liquid extraction for the determination of ciprofloxacin residues in water samples by high performance liquid chromatography–diode array detector

**DOI:** 10.1186/s13065-019-0543-5

**Published:** 2019-03-09

**Authors:** Teshome Gezahegn, Bisratewongel Tegegne, Feleke Zewge, Bhagwan Singh Chandravanshi

**Affiliations:** 1 Department of Chemistry, College of Natural Sciences, Addis Ababa University, P.O. Box 1176, Addis Ababa, Ethiopia; 2 Departement of Chemistry, College of Natural Sciences, Bahir Dar University, P.O. Box 79, Bahir Dar, Ethiopia

**Keywords:** Emerging pollutants, Pharmaceuticals, Ciprofloxacin, Salting-out assisted liquid–liquid extraction, Potable water, Wastewater, High performance liquid chromatography–diode array detector

## Abstract

**Background:**

The occurrence of emerging pollutants like pharmaceuticals and related compounds in the aquatic and terrestrial environments is of increasing concern. Ciprofloxacin is one of the pharmaceuticals which is active against a wide range of bacteria. The main objective of this research is to develop a simple method for the extraction and determination of ciprofloxacin residues in environmental water samples.

**Results:**

A salting-out assisted liquid–liquid extraction (SALLE) method for the determination of ciprofloxacin in water samples by high-performance liquid chromatography with diode array detector (HPLC–DAD) was developed. The calibration curve was linear over the range of 0.1–100 μg/L with coefficient of determination (r^2^) of 0.9976. The limits of detection (LOD) and quantification (LOQ) of the method were 0.075 and 0.25 µg/L, respectively. The reproducibility in terms of relative standard deviation (% RSD) was less than 10%. The applicability of the developed method was investigated by analyzing tap water, bottled mineral water and waste water and demonstrated satisfactory recoveries in the ranges of 86.4–120%.

**Conclusion:**

The method offered a number of features including wide linear range, good recovery, short analysis time, simple operation process and environmental friendly. The developed method can be utilized as an attractive alternative for the determination of ciprofloxacin residues in environmental water matrices.

## Introduction

Pharmaceuticals are molecules designed to produce a therapeutic effect both in human and veterinary. Pharmaceuticals contain active ingredients that have been designed to have pharmacological effects and confer significant benefits to society. However, their continuous large-scale consumption and the subsequent release in the environment can be proven fatal for animals and plants. Pharmaceuticals are considered a class of emerging contaminants that have raised great concern in the last few years [1–3]. They are continuously being released in the environment mainly due to insufficient removal (70–80%) in wastewater treatment plants (WWTPs), whereas the remaining 20–30% is due to other sources of pollution, such as livestock and industrial wastes, hospital effluents and disposal of unused or expired pharmaceuticals [4, 5]. They are present in various water bodies because up to 95% of the dose can be excreted or discharged directly into domestic wastewater [6, 7].

Research has shown that these compounds are not effectively removed during conventional wastewater treatment; therefore they are released into the surface waters, as mixtures of parent compounds, their metabolites and transformation by-products. Some pharmaceuticals can persist in the environment and, either via the food chain or via drinking water, can make their way back to humans, while the properties and fate of metabolites and transformation products are still largely unknown [8, 9].

The occurrence of pharmaceutically active compounds in the environmental water has been confirmed with the concentrations usually range at the μg/L to ng/L range in surface waters [10]. In recent years, pharmaceuticals have received growing attention from environmental and health agencies all over the world owing to recent studies showing the occurrence of pharmaceutical compounds in the environment, especially in water bodies and have become one of the emerging water pollutants [11]. The occurrence of pharmaceuticals in wastewater and environmental samples is highly dependent on local diseases, treatment habits and market profiles, thus, the pollution profile can vary significantly between different countries [12]. Pharmaceuticals in water can have potentially toxic effects on the environment and human [13]. However, pharmaceuticals are not included in the models for the assessment of water quality index [14, 15].

**Fig. 1:**
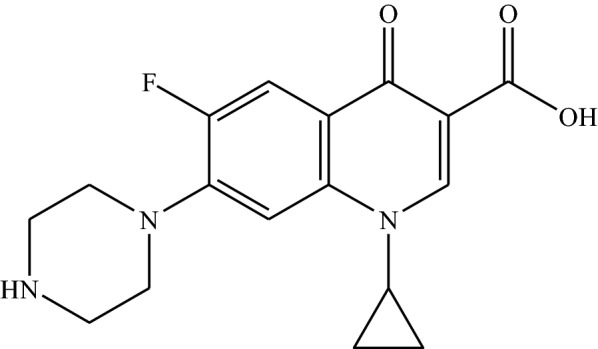
Structure of ciprofloxacin

Antibiotics are among the pharmaceuticals most commonly used in health care systems but the prescription is mostly made on an empirical basis by prescribing broad-spectrum antibiotics [16, 17]. Ciprofloxacin [1-cyclopropyl-6-fluoro-1,4-dihydro-4-oxo-7-(1-piperazinyl)-3-quinolone carboxylic acid] (Fig. 1) is a synthetic fluoroquinolone derivative which has demonstrated broad-spectrum activity against many pathogenic gram-positive bacteria such as *Streptococcus*, *Pneumoniae* and *Enterococcus faecalis*, and gram-negative bacteria including *Salmonella*, *Shigella*, *Campylobacter*, *Neisseria* and *Pseudomonas*. The bacterial action of ciprofloxacin results from interference with enzyme DNA gyrase which is needed for the synthesis of bacterial DNA [18, 19]. It is widely used in the treatment of urinary tract infections, lower respiratory tract infections, bacterial diarrhea, skin and soft tissue infections, bone and joint infections, gonorrhea, and in surgical prophylaxis. Physicians prescribe ciprofloxacin as a first choice of drug [20].

Ciprofloxacin have an amino group (piperazinyl) in the heterocyclic ring and have two dissociation constants. The reported pK_a_ values of ciprofloxacin are 5.76 (acidic) and 8.68 (basic). They are in their zwitterion form in neutral condition and in cationic form in acidic condition [21, 22].

It is very important to have information on the physical and chemical properties of an analyte (e.g., log K_ow_, pK_a_) because that may help to determine whether a compound is likely to concentrate in some specific conditions. Log K_ow_ is an indicator of the lipophilicity of the compound, high log K_ow_ is typical for hydrophobic compounds, whereas a low K_ow_ signifies a compound soluble in water. Most pharmaceuticals have acidic and/or basic functionalities; their ionization rate depends on acidic dissociation constants (i.e. pK_a_ values) and is controlled by solution pH (e.g., pK_a1_ and pK_a2_ values for certain fluoroquinolones (i.e. ciprofloxacin) are in the ranges 5.7 to 6.3 for carboxylic group and 7.6 to 8.3 for protonated amino group, respectively) [23].

Determination of pharmaceuticals in different water samples can be performed by various chromatographic techniques, including HPLC–UV [24], HPLC–DAD [25, 26], LC–MS [27], LC–MS/MS [28, 29] and GC–MS [30, 31]. HPLC is the most common method used for separation and determination of these compounds because most pharmaceuticals are non-volatile [12]. As the residue of pharmaceutical compounds is usually present at very low concentrations in the environmental water, a sample preparation and pre-concentration step are necessary before analysis [23, 32]. Several procedures have been reported for the pre-concentration of pharmaceuticals from water matrices including solid phase extraction (SPE) [27, 33], liquid–liquid extraction (LLE) [34], QuEChERS method [35], magnetic solid phase extraction (MSPE) [36], hollow fiber liquid phase microextraction [37] and salting-out assisted liquid–liquid extraction for non-steroid anti-inflammatory drugs (NSAIDs) [38]. Each of these methods has its own advantages and disadvantages.

Salting-out assisted liquid–liquid extraction [19] is based on the phase separation of water–miscible organic solvents from the aqueous solutions in the presence of high concentration of salts. It uses water–miscible organic solvents which generally have low toxicity as the extractants, and the use of salts causes almost no pollution to the environment [19, 39]. Having such benefits, salting out assisted liquid–liquid extraction was selected to extract ciprofloxacin from water sample in the present study. The objective of this study was the optimization of analytical parameters for the extraction by SALLE and determination of common antibiotic ciprofloxacin residues in water samples using HPLC–DAD.

## Materials and methods

### Chemicals and reagents

All the chemicals used in this study were of analytical grade. Standard ciprofloxacin (99%) was obtained from Addis Pharmaceutical Factory PLC (Ethiopia). HPLC grade methanol (Carlo Erba, Rodano, Italy, HPLC grade, > 99.9%), acetonitrile (Sigma-Aldrich, for HPLC, UV and GC, > 99%), acetic acid (Fisher Chemical UK, 99%), ammonium solution (Fisher Chemical UK, 35%), ethyl acetate (Fine Chem Industries, Mumbai, > 99%), formic acid (Sigma-Aldrich, 85%) and ethanol (Fisher Scientific, UK, 99.9%) were used as received. The different salts used were magnesium sulfate (Fine Chem Industries, Mumbai, 70%), sodium chloride (Sigma-Aldrich, 99.5%), ammonium acetate (BDH Chemical Ltd, England, 96%), and sodium acetate anhydrous (BDH Chemical Ltd, England, 96%). Distilled water was used throughout the study.

### Instrumentation

The HPLC system used in the present study was Agilent 1200 Series equipped with Quaternary Pump, Agilent 1200 Series Vacuum Degasser, Agilent 1200 Series Autosampler and Agilent 1200 Series Diode Array Detector Purchased from Agilent Technologies (Hewlett-Packard Strasse Waldbronn, Germany). Chromatographic separation of the compounds was performed on a C18 analytical column (Techsphere 5ODS, 25 cm × 4.6 mm ID; HPLC Technology, Macclesfield, Cheshire, UK). Data acquisition and processing were accomplished with LC Chemstation software (Agilent Technologies). Adwa pH meter (AD1020 pH/mV/ISE/Temperature, Hungary) was used for the determination of the sample pH and A 800 model centrifuge, China, was used to speed up the phase separation. An electronic balance (Adam Equipment Company, UK) was utilized for weighing the different chemicals involved in the experiments. For the measurement of total dissolved solids (TDS) and electrical conductivity, conductivity meter (Postfach 24 80, Germany) was used.

### Preparation of standard solutions

Stock solutions of the ciprofloxacin (20 µg/mL) were prepared in distilled water and stored at 4 °C. Spiked distilled water samples were prepared with the analyte at a known concentration (0.1 µg/mL) to study the extraction performance of salting-out assisted liquid–liquid extraction under different conditions.

### Extraction procedure

**Scheme 1:**
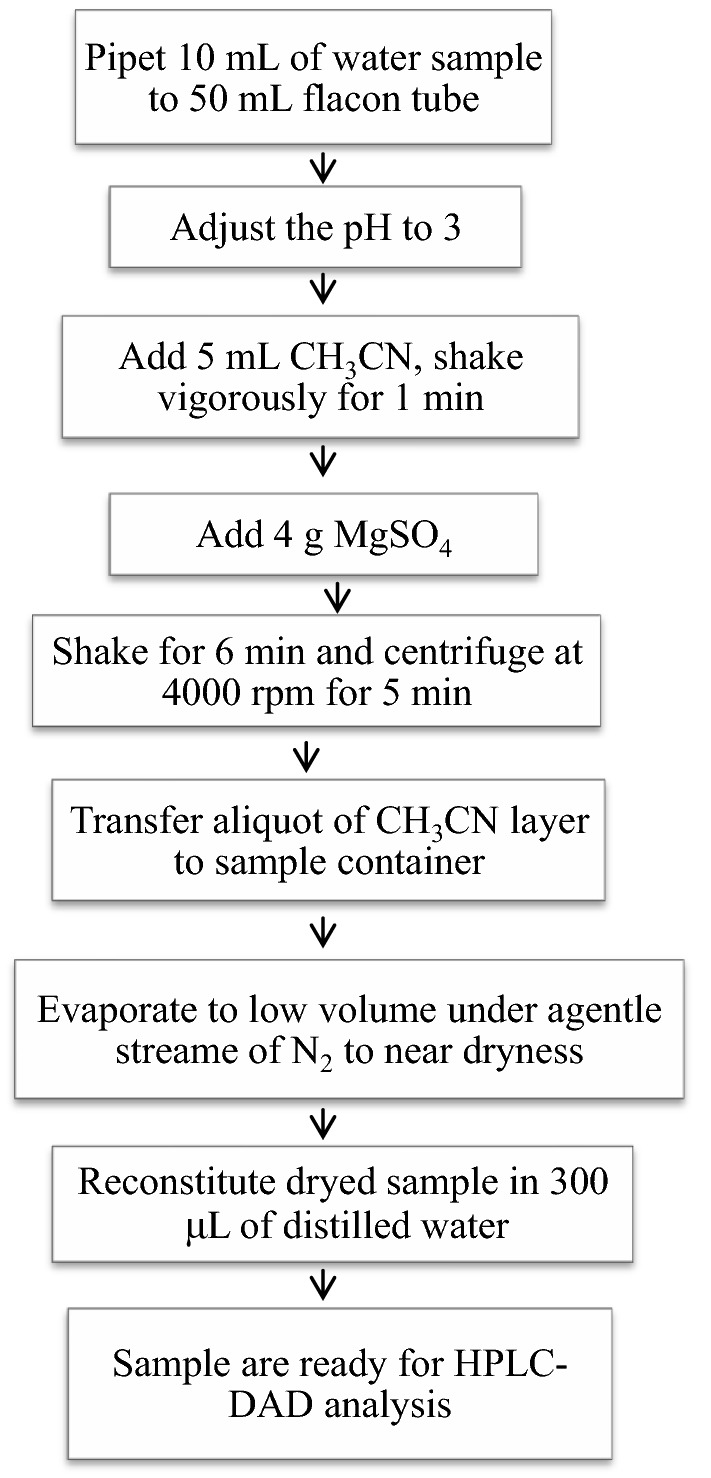
Flow chart of SALLE for the determination of ciprofloxacin

The sample solution (10 mL) was first spiked with a predetermined volume of the standard solution containing the target analyte and quantitatively transferred to each of the 15 mL screw-capped polyethylene test tubes. Then, 5 mL acetonitrile and 4 g MgSO_4_ were added. Thereafter, the solution was shaken gently for 6 min to ensure complete dissolution of the salt. This was followed by centrifugation of the solution at 4000 rpm for 5 min which resulted in phase separation. The upper organic phase was carefully withdrawn using micro-syringe and the extract was dried under a steady stream of nitrogen gas and reconstituted using distilled water (1 mL) and then transferred to a vial for subsequent injection to the HPLC–DAD system. The schematic flow chart of the extraction procedure is given in Scheme 1.

### Method evaluation

The developed SALLE method combined with HPLC–DAD was validated utilizing matrix-matched calibration curves, linearity, detection limits and intra- and inter-day precisions. The intra-day repeatability was studied for three replicate experiments and the inter-day repeatability was investigated for three consecutive days at optimized extraction condition for an aqueous sample containing 0.1 µg/mL of ciprofloxacin. The linearity was investigated over a concentration range by plotting corresponding HPLC peak areas versus concentrations of studied analyte. Limits of detection (LOD) and quantification (LOQ) were calculated at signal to noise ratio of 3 and 10 times, respectively.

### Environmental water samples

Environmental water samples (1 L each) were collected from wastewater samples from two pharmaceutical industries (PIW1 and PIW2), hospital wastewater (HWW), Addis Ababa Sewerage Treatment Plant (AASTP) and river water (RW) in 1 L amber colored glass bottles. Immediately after the arrival of the sample to the laboratory some physic-chemical parameters like electrical conductivity, pH, total dissolved solids (TDS) and salinity of the water samples were examined. Before the experiment, all the water samples were filtered through 0.45 µm filter paper and stored in amber colored bottles at 4 °C in the refrigerator.

### Chromatographic operating conditions

The optimum mobile phase composition utilized throughout the chromatographic analysis was 0.1% formic acid in water/acetonitrile (70:30, v/v) at a flow rate of 0.9 mL/min in isocratic mode. The column temperature was maintained at 35 °C and the detector was adjusted at the optimum detection wavelength of 277 nm with a bandwidth of 4 nm. An aliquot of 20 μL of the extracted sample was injected into the HPLC column automatically and eluted for 10 min run time and 2 min post-time. Finally, the peak area was utilized as an instrumental response and the analysis was obtained under the aforementioned chromatographic conditions.

#### Statistical analysis

All the measurements were done in triplicate. The data were analyzed using statistical software (SPSS Version 21). The results are reported as mean ± SD. Differences were considered significant when p < 0.05. The graphical expression was done using Microsoft excel 7.

## Result and discussion

### Selection of extraction solvent

Five organic solvents namely methanol, acetone, acetonitrile, diethyl ether, and ethyl acetate were examined as extraction solvent. The extraction capabilities of these solvents are depicted in (Table 1). It can be seen that under the same extraction condition, acetonitrile provided the best results with the only peak of the selected analyte. The other solvents used did not show any peak in the retention time of the target analyte (ciprofloxacin) in the chromatogram. The first two solvents, i.e. methanol and acetone are completely miscible with water and they were unable to produce phase separation at all after centrifugation. Hence they were not taken as candidates for further comparison. However, the last two, diethyl ether and ethyl acetate produced the phase separation before the addition of salt. Hence additional comparison steps were carried out for these three solvents which gave clear phase separation. From the three solvents, only acetonitrile showed the peak of the analyte in the chromatogram and it was selected as an extraction solvent. As it was reported [40], acetonitrile is miscible with water in any proportion at room temperature, lowering the temperature or addition of salt significantly reduced the mutual miscibility, even resulting in phase separation of acetonitrile from the aqueous phase. The other two solvents diethyl ether and ethyl acetate did not showed any peak in the chromatogram presumably because they could not extract the analyte due to their lower polarity than acetonitrile.

**Table 1:** Solvents examined for the extraction of ciprofloxacin during analysis by HPLC–DAD

Extraction solvent	Phase separation during extraction	Peak of analyte
Acetonitrile	Partition on the addition of salt	Observed
Acetone	No partition and salt did not dissolved	Not observed
Diethyl ether	Partition before addition of salt	No observed
Ethyl acetate	Partition before addition of salt	No observed
Methanol	No partition and salt did not dissolved	No observed

### Effect of volume of extraction solvent

**Fig. 2:**
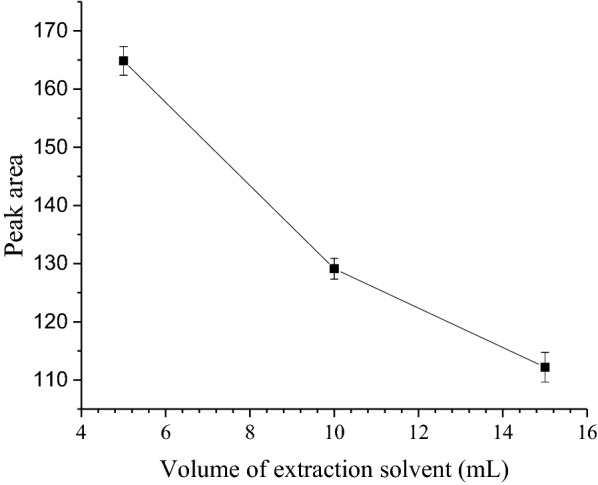
Effect of volume of extraction solvent on the SALLE efficiency

After acetonitrile was selected as the extraction solvent, its volume was optimized using the same extraction condition. The volume of 5, 10 and 15 mL of acetonitrile was used for the selection of the optimum volume for the analyte extraction from 10 mL of sample. As shown in (Fig. 2), maximum peak area was obtained when the volume was 5 mL. When the volume of acetonitrile was below 5 mL, the phase separation was not easy and it was very difficult to take the upper organic phase separately. Similarly, at higher volumes of acetonitrile, above 5 mL, the volumes of the upper organic phase get increased but decreasing the analyte enrichment by dilution and hence further higher volumes were not examined.

### Effect of sample solution pH

**Fig. 3:**
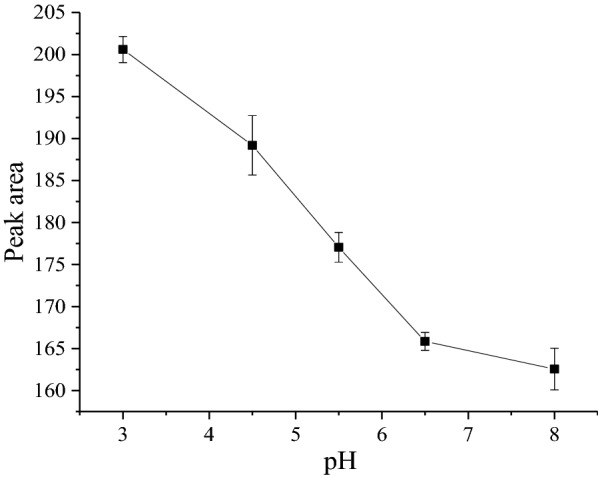
Effects of pH on the SALLE efficiency

The pH value is important as it affects the ionization status as well as the solubility of the analytes [41, 42]. For efficient extraction of ionizable and relatively polar compounds, pH of the sample solution plays a decisive role. The sample solution pH should be lower than the pK_a_ of the analytes to obtain the target analytes in their unionized forms so that they have a higher tendency to partition into the organic phase [43]. The effect of varying pH values of the sample solution on the extraction efficiency was studied in the range of 3.0–8.0. The results are depicted in (Fig. 3), which demonstrated that the extraction efficiency decreased by increasing the pH up to about 8.0. Therefore, the pH value of 3.0 was selected for the extraction of ciprofloxacin.

When a water sample is acidified to a pH that is less than the pK_a_ value of target compounds, the acids are non-ionized which leads to their adsorption through the reversed–phase interactions. At acidic conditions they are in cationic form, which is important for their retention during the extraction. At basic conditions, the anionic species of both acidic and piperazinylic quinolones are less retained in comparison to cationic, zwitterionic and neutral species [43, 44].

### Effect of type and amount of the salt

**Fig. 4:**
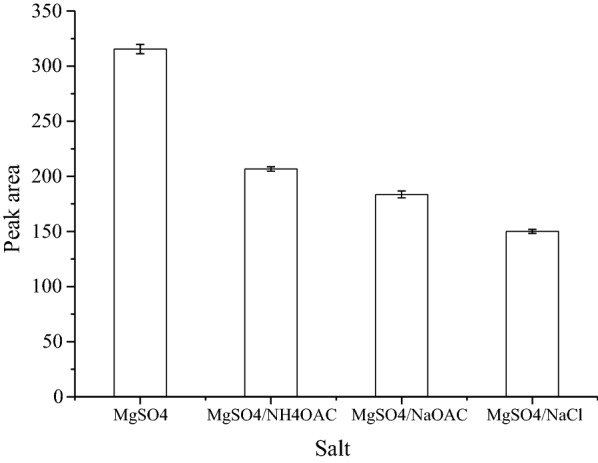
Effect of salt type on the SALLE efficiency

**Fig. 5:**
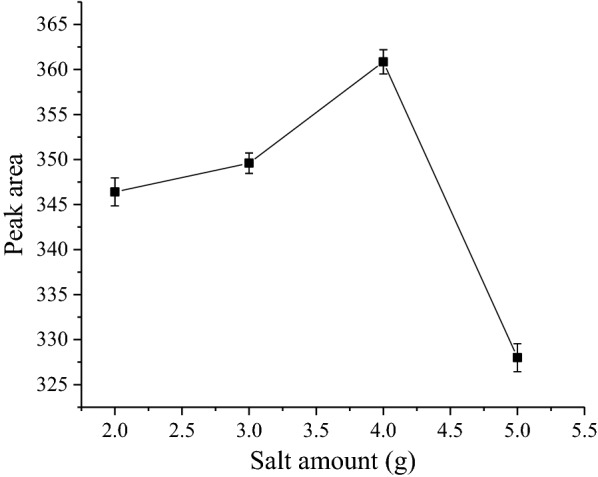
Effect of the salt amount on the SALLE efficiency

Different salts and different salt concentrations cause different degrees of phase separation. The effect of ionic strength was extensively evaluated in traditional liquid–liquid extraction. The addition of a salt is often used to decrease the solubility of hydrophilic compounds in the aqueous phase through a salting-out effect and consequently increase the partition of analytes into the organic phase [19]. In order to obtain phase separation and the optimum extraction efficiency, several salts with different combinations (MgSO_4_, MgSO_4_ with NaCl, MgSO_4_ with NH_4_OAC, and MgSO_4_ with NaOAC) were examined (Fig. 4). The results demonstrated that MgSO_4_ separately provided higher extraction efficiency than the other salts. This may be due to its high ionic strength per unit concentration in the aqueous phase compared to its combination with other salts. It should be pointed out that any strong Lewis base could have interaction with magnesium and impact on the extraction efficiency because magnesium is a strong Lewis acid [40]. Therefore, MgSO_4_ was selected for further study. Meanwhile, the effect of the amount of MgSO_4_ on the extraction efficiency was investigated, varied amounts of MgSO_4_ from 2.0 to 5.0 g (changed every 1.0 g) were added to 15 mL mixed solution (contained 10 mL treated sample solution and 5 mL acetonitrile). It is evident from (Fig. 5) that the optimum amount of MgSO_4_ for the extraction of analysts and the phase separation was considered to be 4 g at room temperature in a 10 mL sample solution. At lower amount of MgSO_4_ the extraction of analyte was not complete while at much higher amount (5 g or more) of MgSO_4_ might resulted to reverse the extraction of analyte due to increase in polarity of the aqueous phase.

### Effect of centrifuge speed

**Fig. 6:**
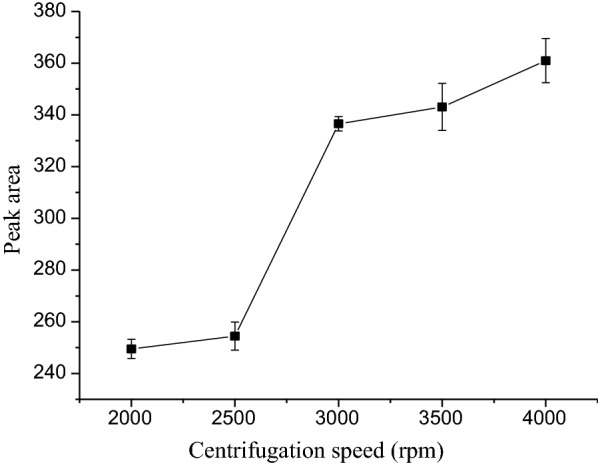
Effect of centrifuge speed on the SALLE efficiency

Centrifugation speed is one of the most important parameters in the sample preparation steps and also plays a key role in the separation of the phases and thus results in a clear solution. In order to obtain the highest signal, the speed was varied from 2000 to 4000 rpm. The corresponding experimental results revealed that the peak areas were increasing with the centrifuge speed, up to 4000 rpm (Fig. 6). Hence 4000 rpm were taken as optimum centrifugation speed. Further higher centrifugation speeds were not examined.

### Extraction time

**Fig. 7:**
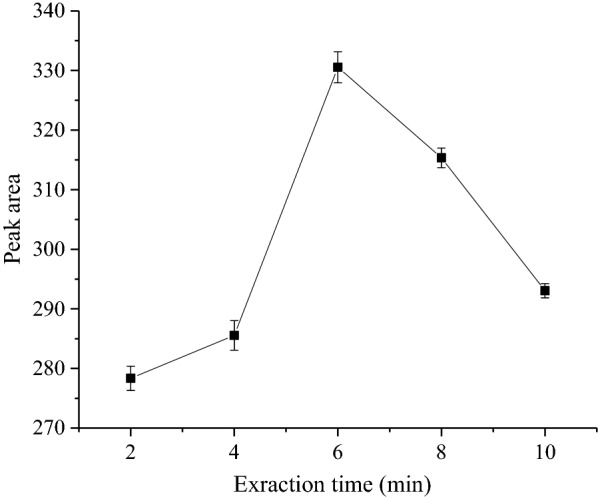
Effect of extraction time on the SALLE efficiency

Mass transfer is a time-dependent process and is also factors in most of the extraction procedures. In the present study, the effect of extraction time on the extraction of analyte was investigated over the range of 2–10 min. The experimental results revealed that 6 min extraction time was found to be optimum. This may be attributed to the very fast mass transfer taking place initially but before the establishment of the equilibrium state, which was achieved later, around 6 min (Fig. 7). Therefore, extraction time of 6 min was found to be the optimum time and used throughout this study. Longer extraction time resulted in decrease of analyte extraction. This might be due to higher miscibility of the two phases at longer extraction (contact) time.

### Selected physicochemical parameters of water samples

Physicochemical parameters are evaluated to determine the degree of contaminants in the water that can affect the water quality and consequently the human health [45]. These are evaluated usually based on scientifically assessed acceptable levels of toxicity to either humans or aquatic organisms [46]. Selected physicochemical parameters of the environmental water samples collected for the determination of ciprofloxacin antibiotic were determined and are presented in Table 2. From all the water samples analyzed, river water was found to have the highest value in conductivity, TDS and salinity than the rest of the water samples. Hospital wastewater showed basic pH and the others are comparably neutral.

**Table 2:** Physicochemical parameters of the environmental water samples

Sample	pH	TDS (mg/L)	Conductivity (µS)	Salinity (0/00)
HWW	8.8	232	485	0.2
RW	7.5	2160	4240	2.2
PIW1	6.8	209	441	0.2
PIW2	6.4	315	656	0.3
AASTP	7.4	543	1128	0.5

*HWW* hospital wastewater, *RW* river water, *PIW* pharmaceutical industry waste, *AASTP* Addis Ababa Sewerage Treatment Plant

### Validation of the method

The analytical characteristics of the proposed method were determined under the optimal conditions. The analyte (ciprofloxacin) showed a single characteristic peak at retention time of about 0.333 min. The calibration curves were established by analyzing the extract of the spiked water sample with the analyte at five different concentration levels. Each level was extracted in triplicate and each extract was analyzed. The calibration curve was obtained by plotting the peak areas versus concentration of the analyte. The results obtained revealed that the calibration curve was linear in the concentration range 0.1–100 µg/L. The coefficients of determination (R^2^) for the analyte was higher than 0.9976, indicating good linearity over the studied concentrations range. The limits of detection (LOD) and quantification (LOQ) were determined as the minimum analyte concentration yielding three and ten times the signal to noise (S/N) ratio, respectively. Thus, LOD was 0.075 µg/L and the LOQ was 0.25 µg/L. Precision was demonstrated by determining the inter- and intra-day relative standard deviation (% RSD) of the analysis with 0.1 µg/mL spiked water sample. The intra-day precision was evaluated by analyzing the spiked samples in the same day and the RSD was found to be 1.7% while inter-day precision was performed for 3 days and the RSD obtained was 6.9% which are in the acceptable range. This further indicates that the short retention time did not make an issue in day to day sample analysis.

### Application to environmental water samples

**Fig. 8:**
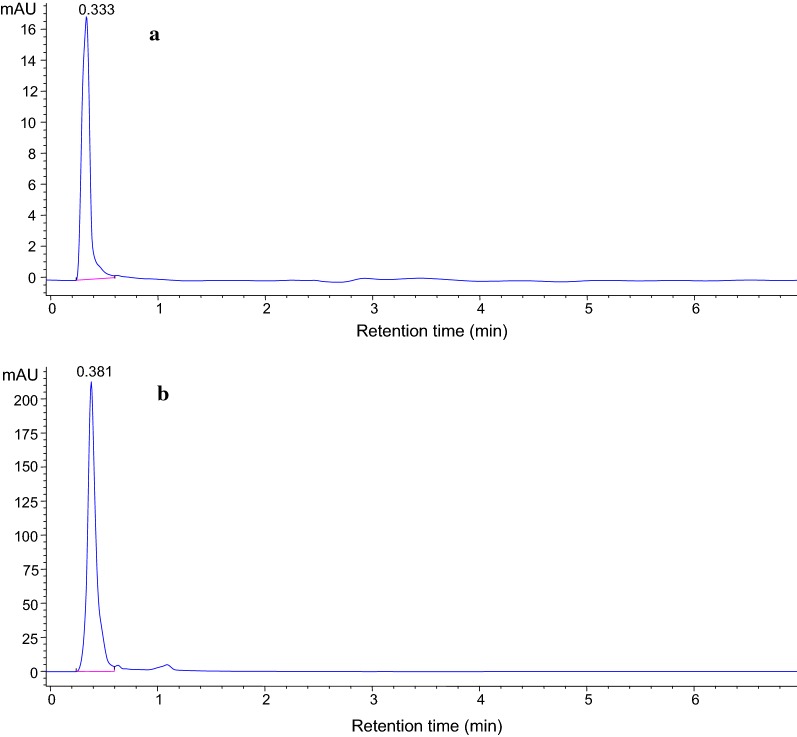
Typical representative chromatogram of **a** unspiked and **b** spiked wastewater. Extraction conditions: 10 mL spiked at 0.1 μg/mL; volume of acetonitrile, 5 mL; amount of salt added, 4 g MgSO_4_; extraction time, 6 min; pH of the sample solution, 3; centrifugation speed, 4000 rpm; n = 3

In order to investigate the applicability of the proposed SALLE method, recovery experiments were carried out on four kinds of water samples of different origin spiked with different concentration of ciprofloxacin. The recovery results as shown in Table 3 ranged from 86.4 to 120%. Ciprofloxacin was not detected in tap water, bottled mineral water, and hospital wastewater. It was detected only in the waste water from Addis Ababa Sewage Treatment Plant (AASTP). This could be due to either the water samples analyzed other than wastewater from AASTP, were free from the residues of target pharmaceutical or contained concentrations below the detection limits. The obtained ciprofloxacin concentration in the wastewater sample from sewerage treatment plant was 0.83 µg/mL. This might be because the sewerage treatment plant collects wastes from many toilets from the city. Like many studies, result reveals that most pharmaceuticals found in the wastewater as parent compound and/or its metabolites via excretion, mainly in urine (55–80%) and to a lesser extent in feces (4–30%) [47, 48]. Typical chromatograms of the non-spiked wastewater and spiked wastewater (0.1 µg/mL) samples from Addis Ababa Sewage Treatment Plant using the optimized SALLE–HPLC technique are shown in Fig. 8.

**Table 3:** Recovery results of different water matrices

Types of sample	Spiked level (μg/mL)	% RSD	Recovery (%)
Distilled water	0	–	–
	2.5	0.79	101
	5	2.47	103
Tap water	0	–	–
	0.02	2.78	100
	0.1	5.50	120
	2.5	0.66	86.4
Bottled water	0	–	–
	0.1	1.27	108
	2.5	1.82	91.2
Hospital wastewater	0	–	–
	2.5	5.24	117

### Comparison of present method with the other methods reported in literature

The important analytical parameters of the proposed SALLE–HPLC–DAD method for the determination of ciprofloxacin antibiotic residue were compared to some of the previously reported methods and the data are summarized in Table 4. The proposed method has good and comparable analytical results compared with other methods for extraction and determination of ciprofloxacin in different matrices. Based on the experimental findings the proposed technique has wider linear range and lower limit of detection and quantification compared to others work [49, 50]. In addition, the present method has better values of correlation coefficient (R^2^) and better recovery than the most of the reported methods [35, 51]. However, the present method has higher detection limit and less sensitive than the reported methods based on mass spectrometry detector. But the present method has advantage of easy of operation in contrast to other HPLC/MS methods. Therefore, the proposed SALLE–HPLC–DAD can be considered as one of the preferred alternative having a promising future for selective and quantitative extraction of pharmaceutical residue contaminating various environmental water systems.

**Table 4:** Comparison between the proposed SALLE–HPLC–DAD method and some reported methods for the determination of ciprofloxacin in deferent matrices

Analytical method	Linear range µg/L	R^2^	LOD µg/L	LOQ µg/L	% Recovery	Refs.
QuEChERS–LC–MS/MS	0.01–10	0.9951	0.033	0.1	73–125	[35]
SPE–HPLC–MS	10–1000	0.9981	–	0.04	89–97	[50]
SPE–LC–MS/MS	0.5–600	0.9935	0.001	0.003	78–98	[52]
SPE–LC–MS/MS	0.01–10	0.9968	0.001	0.01	47–117	[51]
SPE–HPLC–DAD	0.5–20	0.9991	0.25	0.5	90–110	[49]
SALLE–HPLC–DAD	0.1–100	0.9976	0.075	0.25	86–120	This work

## Conclusion

A SALLE method coupled with HPLC–DAD was developed and successfully utilized for the determination of ciprofloxacin residue in environmental water. The method offered a number of features including wide linear range, high recovery, and short analysis time, simple operation process and environmentally friendly. Based on the present findings SALLE, coupled with a water–miscible extraction solvent, acetonitrile, and MgSO_4_ as the salting-out agent, could be taken as a significantly promising extraction and pre-concentration method for trace analysis of water-soluble pharmaceutical (ciprofloxacin) residues which are difficult to be extracted with non-polar organic solvents from various environmental water samples. The method has advantages of simplicity, easy operation and short analysis time with consumption of low volume of the less hazardous organic solvent, acetonitrile. Therefore, the developed method can be utilized as an attractive alternative for the determination of antibiotic ciprofloxacin in environmental water matrices.

## Abbreviations

AASTP: Addis Ababa Sewerage Treatment Plant
GC–MS: gas chromatography–mass spectrometry
HPLC–DAD: high-performance liquid chromatography with diode array detector
HPLC–UV: high-performance liquid chromatography with ultraviolet detector
HWW: hospital wastewater
LC–MS: liquid chromatography–mass spectrometry
LC–MS/MS: liquid chromatography–mass spectrometry/mass spectrometry
LLE: liquid–liquid extraction
LOD: limit of detection
LOQ: limits of quantification
MSPE: magnetic solid phase extraction
NSAIDs: non-steroid anti-inflammatory drugs
PIW1: pharmaceutical industries wastewater one
PIW2: pharmaceutical industries wastewater two
QuEChERS: Quick Easy Cheap Effective Rugged Safe
QuEChERS–LC–MS/MS: Quick Easy Cheap Effective Rugged Safe–liquid chromatography mass spectrometry/mass spectrometry
RSD: relative standard deviation
RW: river water
SALLE: salting-out assisted liquid–liquid extraction
SALLE–HPLC–DAD: salting-out assisted liquid–liquid extraction–high-performance liquid chromatography with diode
SPE: solid phase extraction
SPE–HPLC–DAD: solid phase extraction–high-performance liquid chromatography with diode array detector
SPE–HPLC–MS: solid phase extraction-high-performance liquid chromatography–mass spectrometry
SPE–LC–MS/MS: solid phase extraction–high-performance liquid chromatography–mass spectrometry/mass spectrometry
TDS: total dissolved solids
WWTPs: wastewater treatment plants

## References

[1] Khan A, Khuda F, Elseman AM, Aly Z, Wang X (2018). Innovations in graphene-based nanomaterials in the pre-concentration of pharmaceuticals waste. Environ Technol Rev.

[2] Ebele AJ, Abdallah MA, Harrad S (2017). Pharmaceuticals and personal care products (PPCPs) in the fresh water aquatic environment. Emerg Contaminants.

[3] WHO (2012). Pharmacutical in drinking water.

[4] Lin AY, Yu T, Lin C (2008). Pharmaceutical contamination in residential, industrial, and agricultural waste streams: risk to aqueous environments in Taiwan. Chemosphere.

[5] Logarinhoa F, Rosadoa T, Lourenc C, Barrosod M, Araujoc AR, Gallardo E (2016). Determination of antipsychotic drugs in hospital and wastewater treatment plant samples by gas chromatography/tandem mass spectrometry. J Chromatogr B.

[6] Bottoni P, Caroli S, Caracciolo AB (2010). Pharmaceuticals as priority water contaminants. Toxicol Environ Chem.

[7] Baranowska I, Kowalski B (2012). A rapid UHPLC method for the simultaneous determination of drugs from different therapeutic groups in surface water and wastewater. Bull Environ Contam Toxicol.

[8] Fatta D, Achilleos A, Nikolaou A, Meric S (2007). Analytical methods for tracing pharmaceutical residues in water and wastewater. Trends Anal Chem.

[9] Jones OA, Lester JN, Voulvoulis N (2005). Pharmaceuticals: a threat to drinking water?. Trends Biotechnol.

[10] Tran NH, Urase T, Ta T (2014). A Preliminary study on the occurrence of pharmaceutically active compounds in hospital wastewater and surface water in Hanoi. Vietnam. Clean Soil Air Water.

[11] Kumar A, Xagoraraki I (2010). Pharmaceuticals, personal care products and endocrine-disrupting chemicals in U.S. surface and finished drinking waters: a proposed ranking system. Sci Total Environ.

[12] Salgado R, Noronha JP, Oehmen A, Carvalho G, Reis MA (2010). Analysis of 65 pharmaceuticals and personal care products in 5 wastewater treatment plants in Portugal using a simplified analytical methodology. Water Sci Technol.

[13] Alshakka M, Ibraim MI, Hassali M (2016). Hazards of pharmaceuticals in water as new area in eco-pharmacovigilance research. J Pharm Pract Community Med.

[14] Mohammed SI, Abdulrazzaq KA (2018). Developing water quality index to assess the quality of the drinking water. Civil Eng J.

[15] Khudair BH, Jasim MM, Alsaqqar AS (2018). Artificial neural network model for the prediction of groundwater quality. Civil Eng J.

[16] Getachew E, Aragaw S, Adissie W, Agalu A (2013). Antibiotic prescribing pattern in a referral hospital in Ethiopia. Afr J Pharm Pharmacol Therap Part B Gen Syst Pharmacol.

[17] Worku F, Tewahido D (2018). Retrospective Assessment of Antibiotics Prescribing at Public Primary Healthcare Facilities in Addis Ababa, Ethiopia. Interdiscip Perspect Infect Dis.

[18] Varak M, Ebrahimi M (2018). Preconcentration and determination of ciprofloxacin with solid-phase microextraction and silica-coated magnetic nanoparticles modified with salicylic acid by UV-Vis spectrophotometry. Eurasian J Anal Chem.

[19] Xia Q, Yang Y, Liu M (2012). Aluminium sensitized spectrofluorimetric determination of fluoroquinolones in milk samples coupled with salting-out assisted liquid–liquid ultrasonic extraction. Spectrochim Acta Part A Mol Biomol Spectros.

[20] Olaitan OJ, Anyakora C, Bamiro T, Tella AT (2014). Determination of pharmaceutical compound in surface and underground water by solid phase extraction–liquid chromatography. J Environ Chem Toxicol.

[21] Gros M, Mozaz S, Barcelo D (2013). Rapid analysis of multiclass antibiotic residues and some of their metabolites in hospital, urban wastewater and river water by ultra-high performance liquid chromatography coupled to quadrupole-linear ion trap tandem mass spectrometry. J Chromatogr A.

[22] Khattab F, Salem H, Riad S, Elbalkiny H (2014). Determination of fluoroquinolone antibiotics in industrial waste water by high-pressure liquidchromatography and thin layer chromatography–densitometric methods. J Planar Chromatogr.

[23] Pavlovic DM, Babic S, Alka J, Horvat M, Macan MK (2007). Sample preparation in analysis of pharmaceuticals. Trends Anal Chem.

[24] Silva DC, Oliveira CC (2018) Development of micellar HPLC-UV method for determination of pharmaceuticals in water samples. J Anal Methods Chem 2018. Article ID 9143730. 10.1155/2018/9143730 10.1155/2018/9143730 PMC5852859 29686934

[25] Madureira TV, Rocha MJ, Cass QB, Tiritan ME (2010). Development and optimization of a HPLC–DAD method for the determination of diverse pharmaceuticals in estuarine surfacewaters. J Chromatogr Sci.

[26] Baranowska I, Kowalski B (2011). Using HPLC method with DAD detection for the simultaneous determination of 15 drugsin surface water and wastewater. Polish J Environ Stud.

[27] Iglesias A, Nebot C, Vázquez B, Coronel-Olivares C, Abuín F, Cepeda A (2014). Monitoring the presence of 13 active compounds in surface water collected from rural areas in Northwestern Spain. Int J Environ Res Public Health.

[28] Ort C, Lawrence MG, Reungoat J, Eaglesham G, Carter S, Keller J (2010). Determining the fraction of pharmaceutical residues in wastewater originating from a hospital. Water Res.

[29] Chen Y, Vymazal J, Březinová T, Koželuh M, Kule L, Huang J, Chen Z (2016). Occurrence, removal and environmental risk assessment of pharmaceuticals and personal care products in rural wastewater treatment wetlands. Sci Total Environ.

[30] Farré M, Petrovic M, Barceló D (2007). Recently developed GC/MS and LC/MS methods for determining NSAIDs in water sample. J Anal Bioanal Chem.

[31] Qureshi T, Memon N, Memon SQ, Shaikh H (2014) Determination of ibuprofen drug in aqueous environmental samples by gas chromatography–mass spectrometry without derivatization. Am J Modern Chromatogr 1(1):45–54. https://www.researchgate.net/publication/283122628. Accessed 13 Jan 2019

[32] Wille K, Brabander HF, De Wulf E, Caeter PV, Janssen CR, Vanhaecke L (2012). Coupled chromatographic and mass-spectrometric techniques for the analysis of emerging pollutants in the aquatic environment. Trends Anal Chem.

[33] Al-Qaim FF, Abdullah P, Othman MR, Latip J, Afiq WM (2013). Development of analytical method for detection of some pharmaceuticals in surface water. Trop J Pharm Res.

[34] Padrón ET, Afonso-Olivares C, Sosa-Ferrera Z, Santana-Rodríguez J (2014). Microextraction techniques coupled to liquid chromatography with mass spectrometry for the determination of organic micropollutants in environmental water samples. Molecules.

[35] Kachhawaha AS, Nagarnaik PM (2017). Optimization of a modified QuEChERS method for multiresidue analysis of pharmaceuticals and personal care products in sewage and surface water by LC-MS/MS. J AOAC Int.

[36] Tolmacheva VV, Apyari VV, Furletov AA, Dmitrienko SG, Zolotov YA (2016). Facile synthesis of magnetic hypercrosslinked polystyrene and its application in the magnetic solid-phase extraction of sulfonamides from water and milk samples before their HPLC determination. Talanta.

[37] Sharifi V, Abbasi A, Nosrati A (2016). Application of hollow fiber liquid phase microextraction and dispersive liquideliquid microextraction techniques in analytical toxicology. J Food Drug Anal.

[38] Noche G, Laespadab ME, Pavónb JL, Corderob BM, Lorenzoa SM (2011). In situ aqueous derivatization and determination of non-steroidalanti-inflammatory drugs by salting-out-assisted liquid–liquid extraction and gas chromatography–mass spectrometry. J Chromatogr A.

[39] Alshishania A, Salhimib SM, Saada B (2018). Salting-out assisted liquid-liquid extraction coupled with hydrophilic interaction chromatography for the determination of biguanides in biological and environmental samples. J Chromatogr B.

[40] Zhang J, Wiley J, Wu H, Kima E, El-Shourbagya TA (2009). Salting-out assisted liquid/liquid extraction with acetonitrile: a new high throughput sample preparation technique for good laboratory practice bioanalysis using liquid chromatography–mass spectrometry. Biomed Chromatogr.

[41] Valente IM, Goncalves LM, Rodrigues JA (2013). Another glimpse over the salting-out assisted liquid–liquid extraction in acetonitrile/water mixtures. J Chromatogr A.

[42] Atlabachew M, Chandravanshi BS, Redi-Abshiro M (2017). Preparative HPLC for large scale isolation, and salting-out assisted liquid-liquid extraction based method for HPLC-DAD determination of khat (*Catha edulis* Forsk) alkaloids. Chem Cent J.

[43] Bedassa T, Megersa N, Gure A (2017). Salting-out assisted liquid-liquid extraction for the determination of multiresidue pesticides in alcoholic beverages by high performance liquid chromatography. Sci J Anal Chem.

[44] Kummerer K (2009). Antibiotics in the aquatic environment-Areview-part I. Chemosphere.

[45] Rahmanian N, Ali SHB, Homayoonfard M, Ali NJ, Rehan M, Sadef Y, Nizami AS (2015) Analysis of physiochemical parameters to evaluate the drinking water quality in the state of perak, Malaysia. J Chem 2015. Article ID 716125. 10.1155/2015/716125

[46] Alemu T, Mulugeta E, Tadese M, Kakaei K (2017). Determination of physicochemical parameters of “Hora” natural mineral water and soil in Senkele Kebele, Oromia Region, Ethiopia. Cogent Chem.

[47] Verlicchi P, Galletti A, Petrovic M, Barcelo D, Al Aukidy M, Zambello E (2013). Removal of selected pharmaceuticals from domestic wastewater in an activated sludge system followed by a horizontal subsurface flow bed—analysis of their respective contributions. Sci Total Environ.

[48] Aukidy AM, Verlicchi P, Voulvoulis N (2014). A framework for the assessment of the environmental risk posed by pharmaceuticals originating from hospital effluents. Sci Total Environ.

[49] Ašperger D, Tišler V, Zrnčić M, Pavlović DM, Babić S, Horvat AJ, Kaštelan- Macan M (2014). HPLC–DAD–FLD determination of veterinary pharmaceuticals in pharmaceutical industry wastewater with precolumn derivatization using fluorescamine. Chromatographia.

[50] Wei R, Ge F, Chen M, Wang R (2012). Occurrence of ciprofloxacin, enrofloxacin, and florfenicol in animal wastewater and water resources. J Environ Quality.

[51] Babic S, Pavlovic MD, Asperger D, Perisa M, Zrncic M, Horvat AJ, Kastelan-Macan M (2010). Determination of multi-class pharmaceuticals in wastewater by liquid chromatography-tandem mass spectrometry (LC-MS-MS). J Anal Bioanal Chem.

[52] Afonso-Olivares C, Torres-Padrón E, Sosa-Ferrera Z, Santana-Rodríguez J (2013). Assessment of the presence of pharmaceutical compounds in seawater samples from coastal area of Gran Canaria Island (Spain). Antibiotics.

